# Supplementary material for: BzAM terpolymer series with stepwise systematic change in hydrophobicity provides a nuanced platform for GPCRs functional studies in native-like nanodiscs
Source: Biosci Rep. 2026 Nov 12;46(11):BSR20260503. doi: 10.1042/BSR20260503 (PMC13768144; doi:10.1042/BSR20260503)
Supplement: Supplementary file 1 [file BSR-2026-0503_supp.pdf]

SUPPLEMENTARY INFORMATION

A:

Soluble fraction n=1

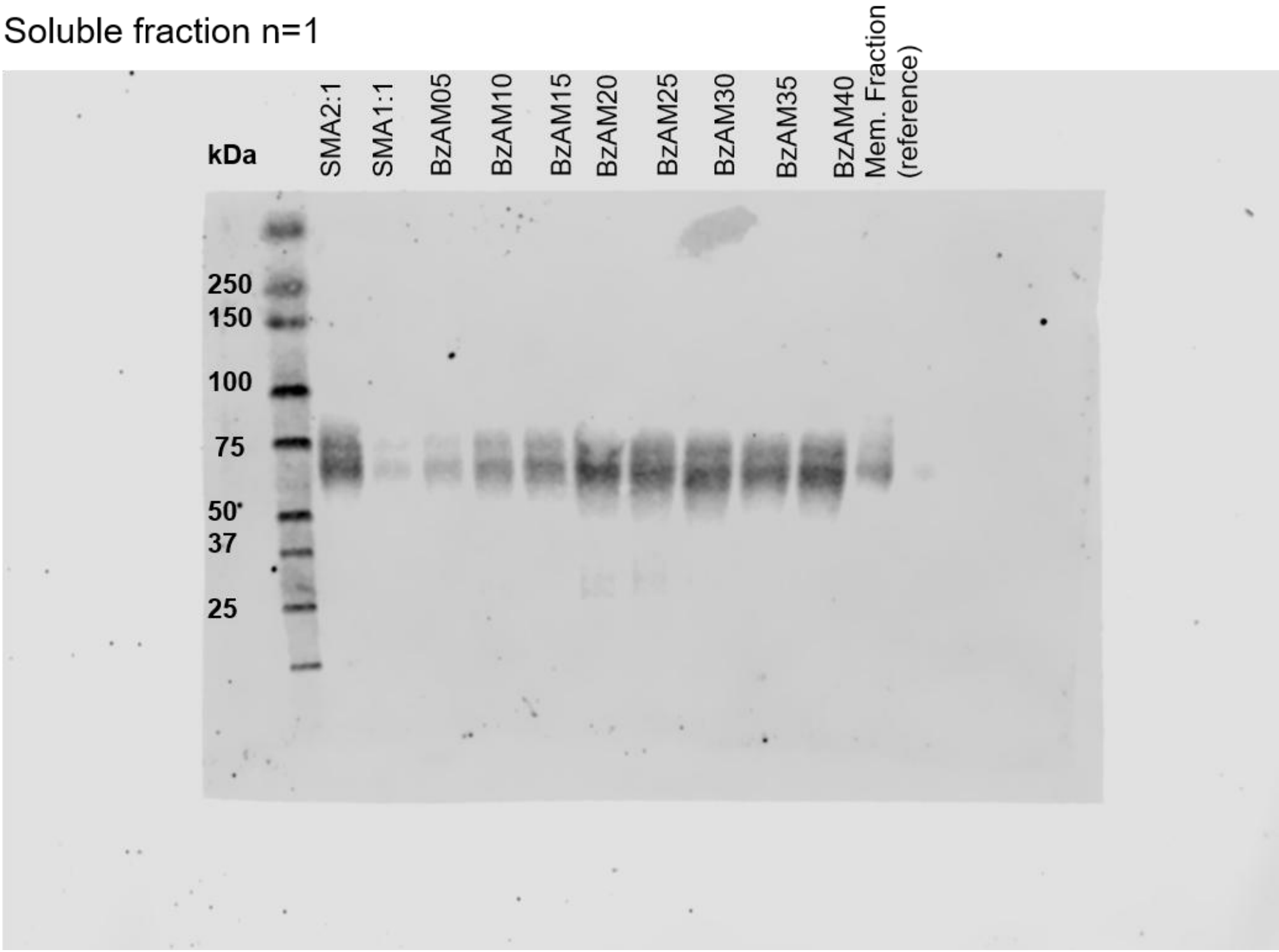

Soluble fraction n=2

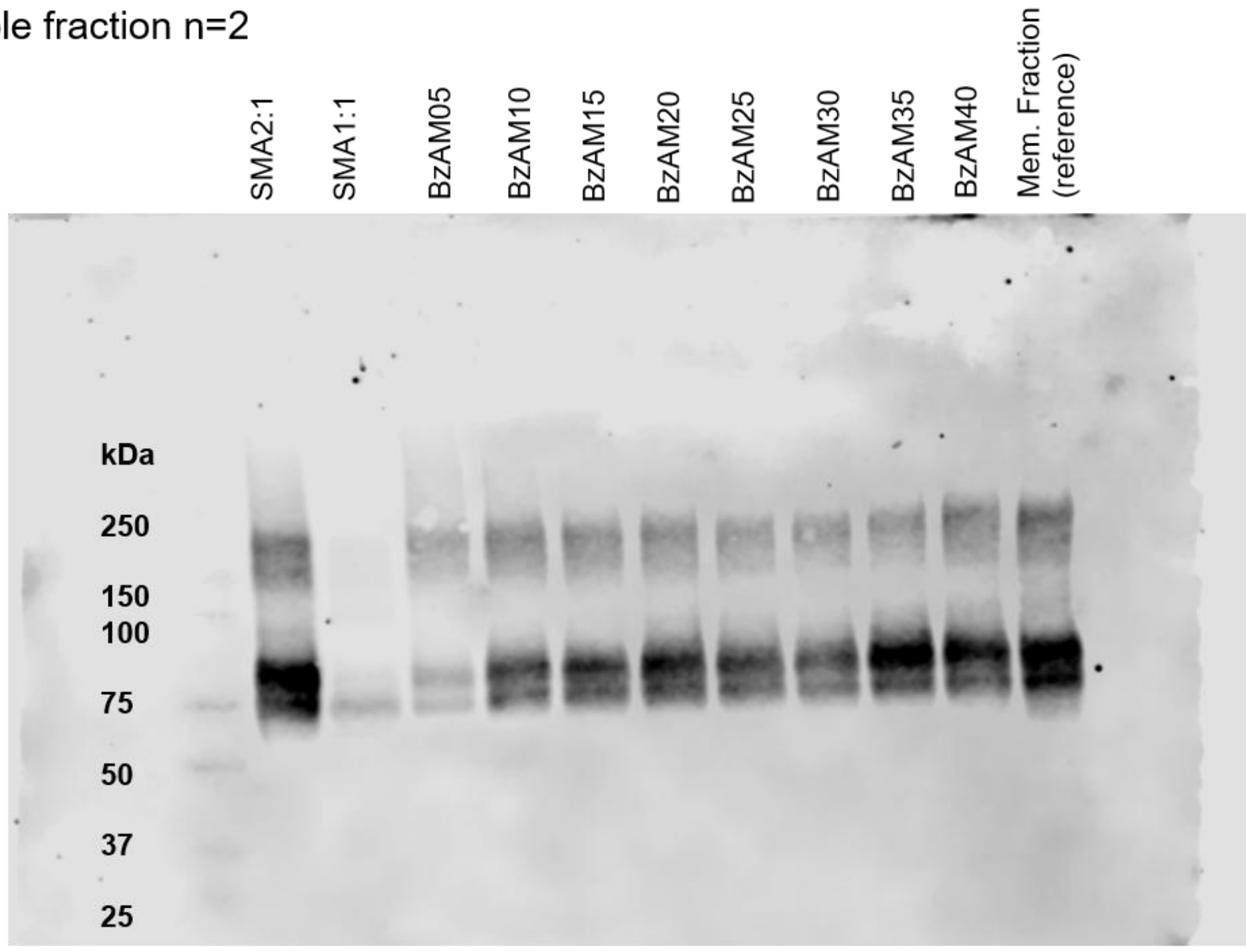

Soluble fraction n=3

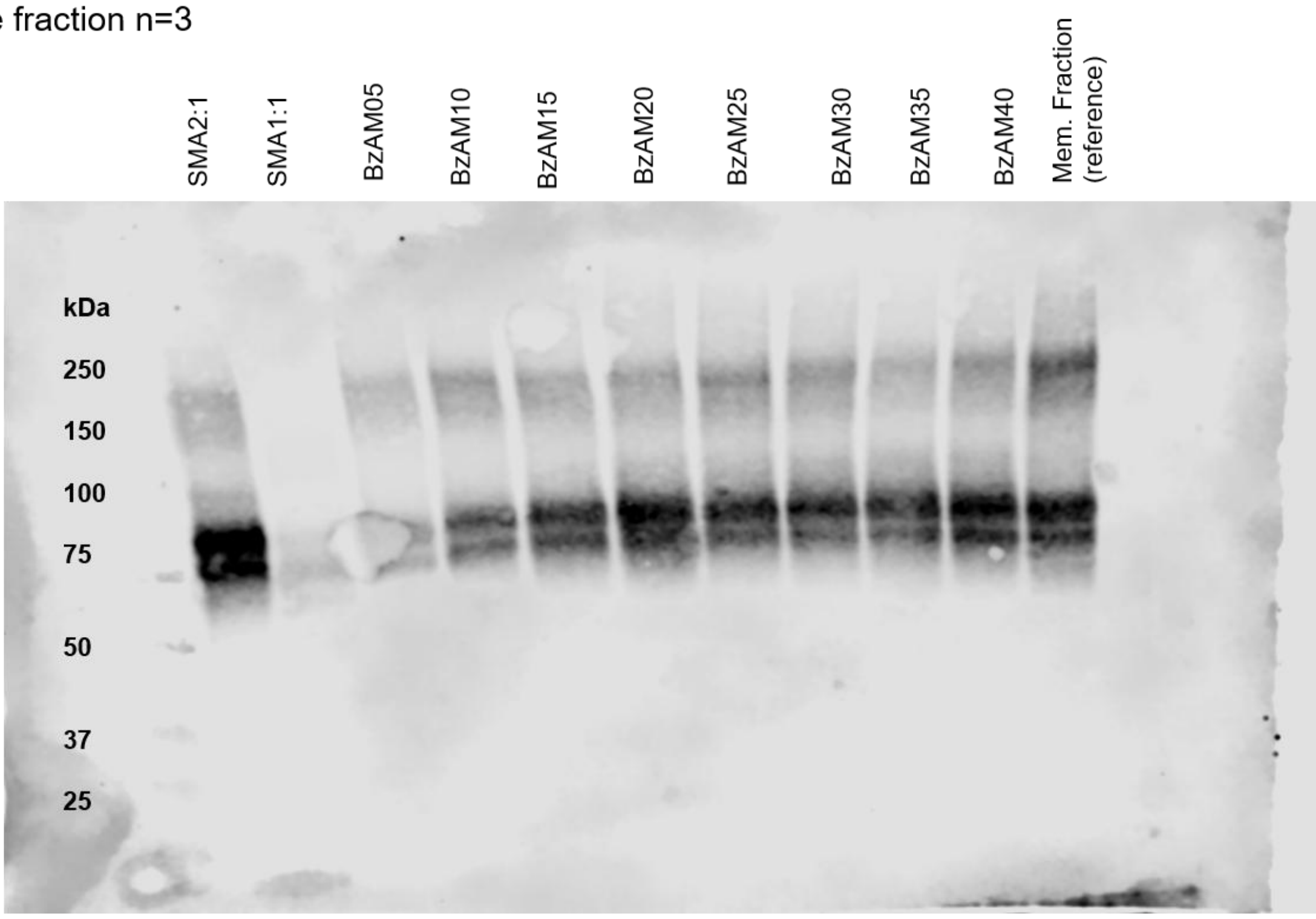

B:

Insoluble fraction n=1

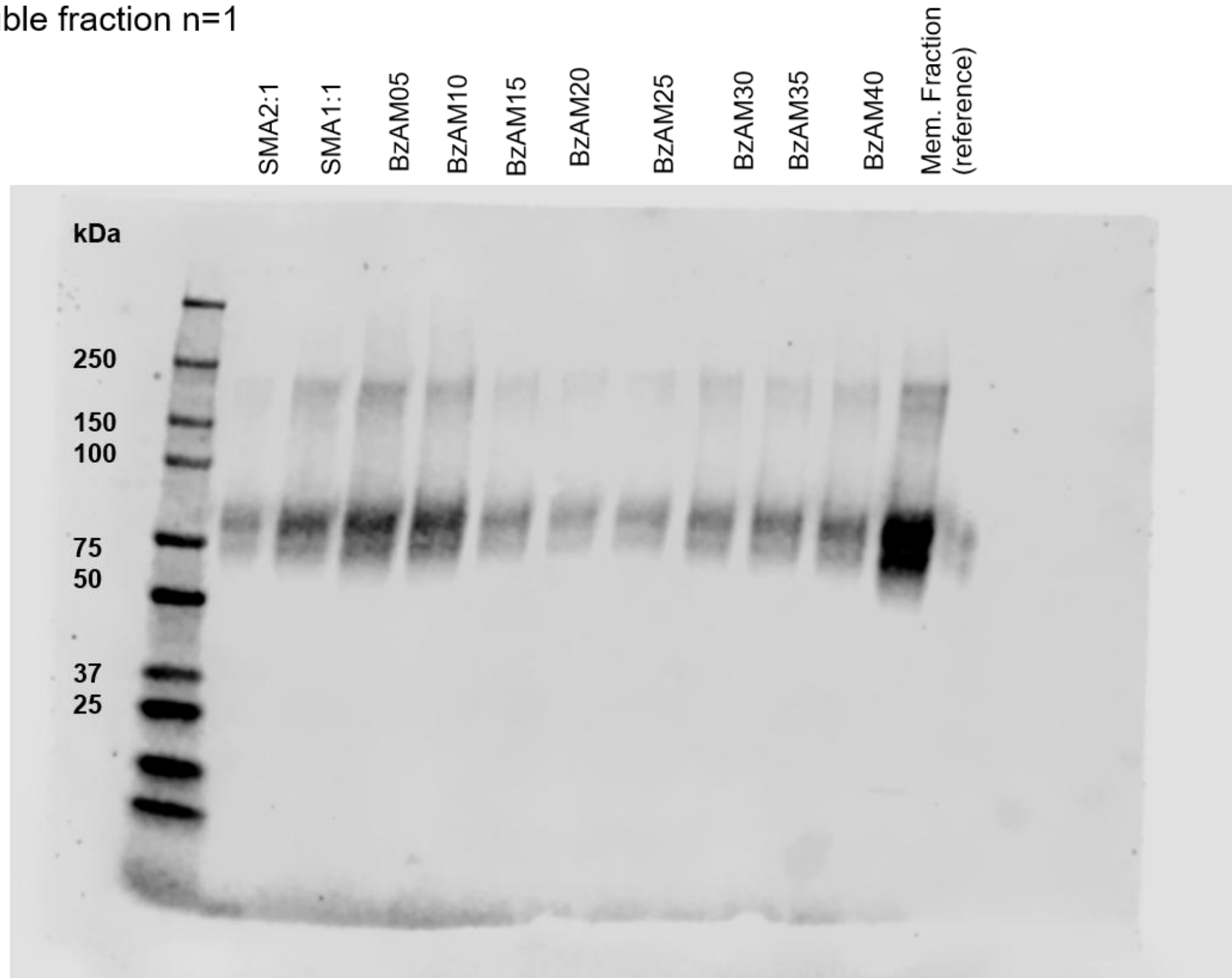

Insoluble fraction n=2

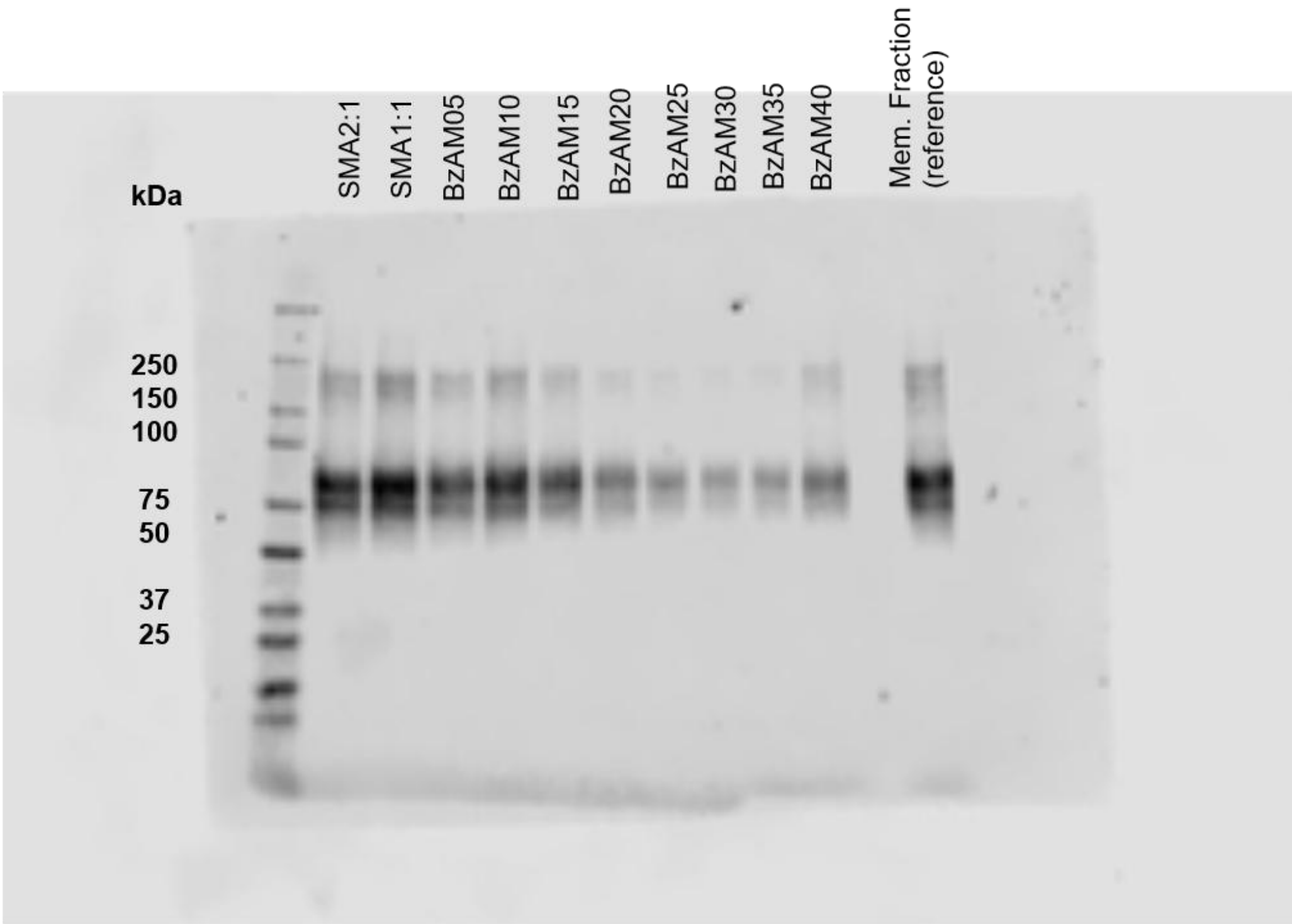

Insoluble fraction n=3

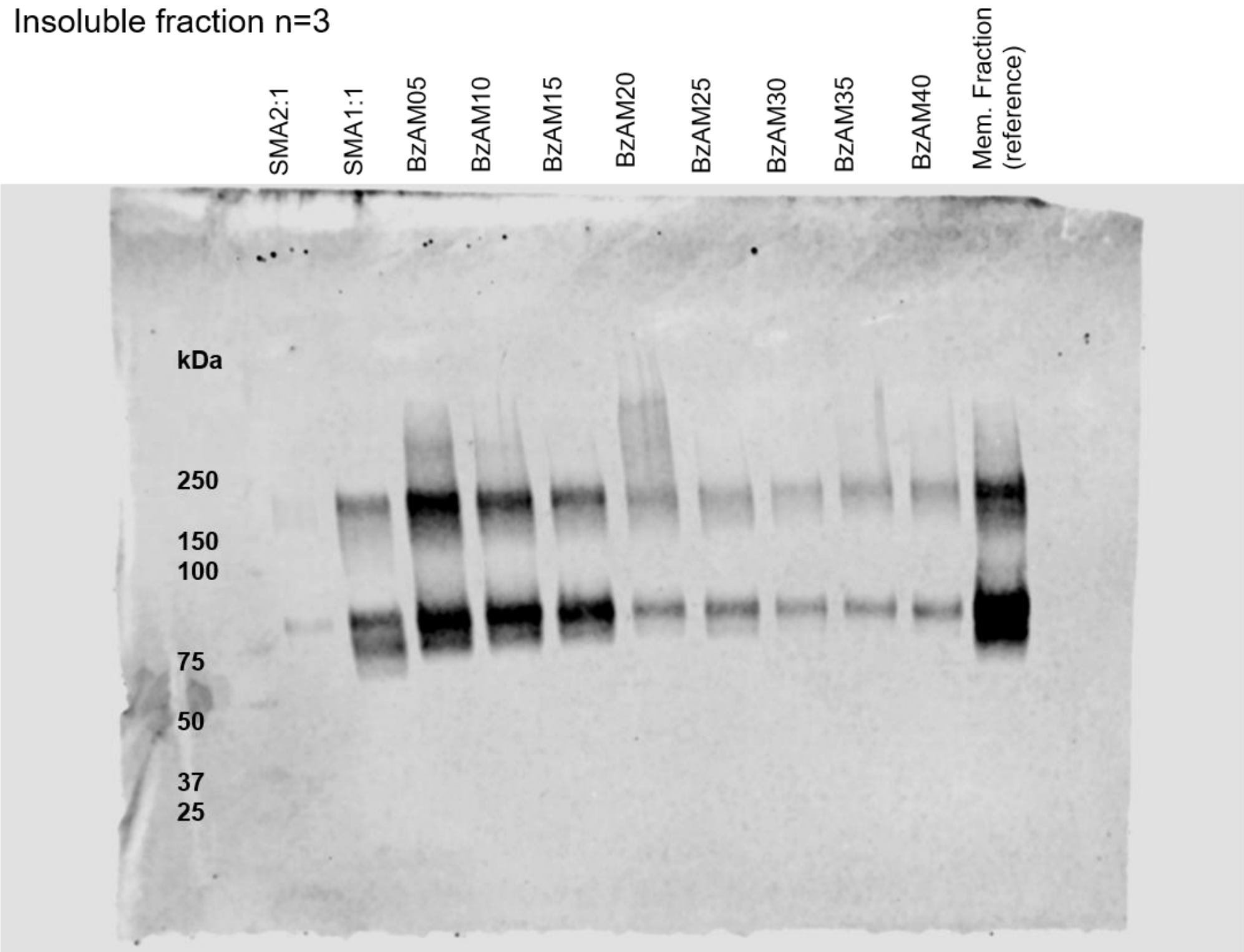

Supplementary Figure 1: Full western blot images (n=3) corresponding to A) Figure 2A and B) Figure 2B. Membrane fraction (reference) denotes the loading control.

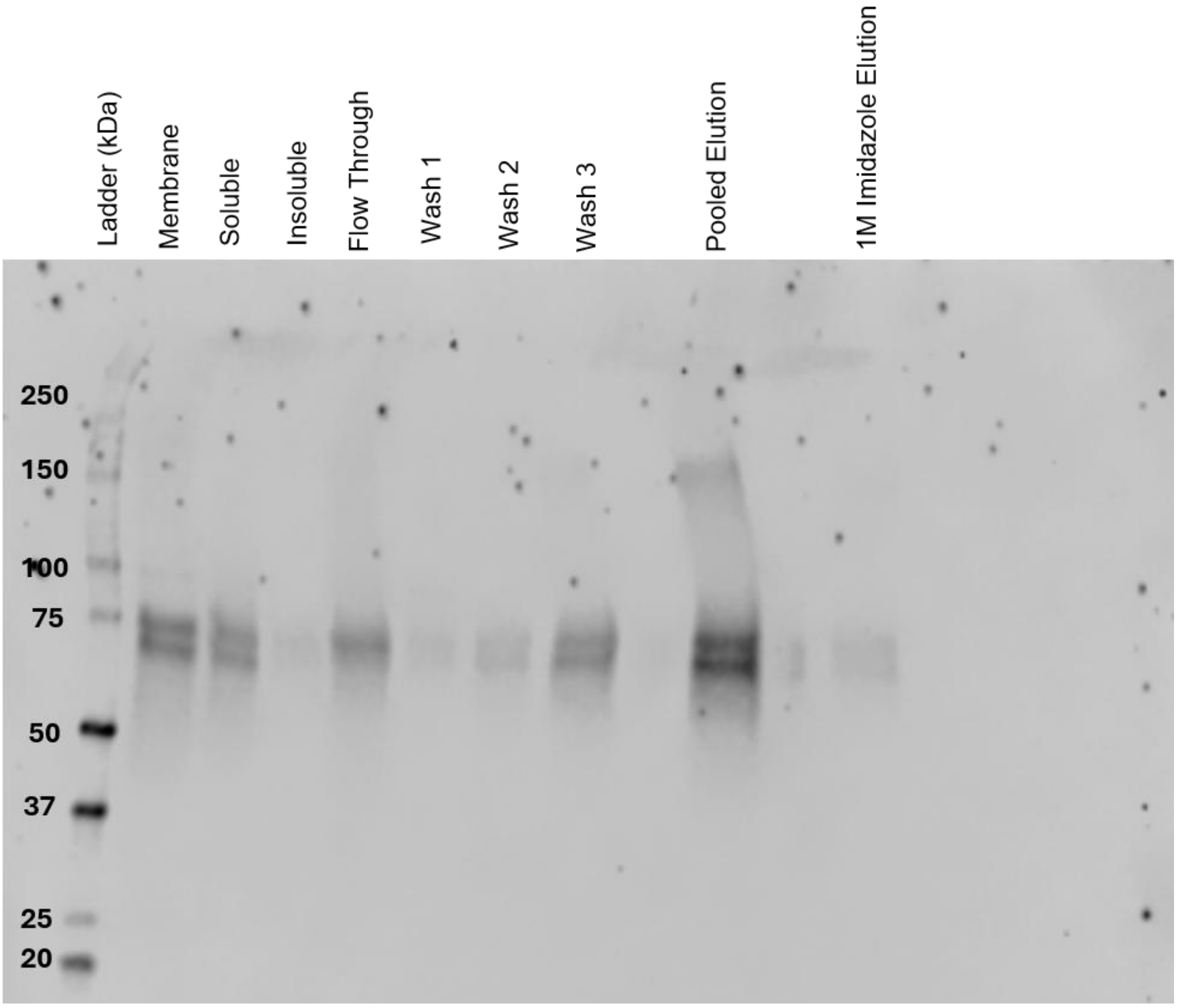

Supplementary Figure 2: Full western blot image corresponding to Figure 5A. Membrane denotes the loading control.
